# CombiFlow: Flow cytometry-based identification and characterization of genetically and functionally distinct AML subclones

**DOI:** 10.1016/j.xpro.2021.100864

**Published:** 2021-09-27

**Authors:** Roos Houtsma, Shanna M. Hogeling, Jan Jacob Schuringa

**Affiliations:** 1 Department of Hematology, University Medical Center Groningen, University of Groningen, Hanzeplein 1, 9713GZ Groningen, the Netherlands

**Keywords:** Cancer, Cell isolation, Flow Cytometry/Mass Cytometry, Stem Cells

## Abstract

Many cancers, including leukemias, are dynamic oligoclonal diseases. Tools to identify and prospectively isolate genetically distinct clones for functional studies are needed. We describe our CombiFlow protocol, which is a combinatorial flow cytometry-based approach to identify and isolate such distinct clones. CombiFlow enables the visualization of clonal evolution during disease progression and the identification of potential relapse-inducing cells at minimal residual disease (MRD) time points. The protocol can be adapted to various research questions and allows functional studies on live sorted cell populations.

For complete details on the use and execution of this protocol, please refer to de Boer et al. (2018).

## Highlights

• Subclones can be identified and sorted based on aberrant marker expression
• Functional in vitro and in vivo studies can be performed on identified subclones
• Combiflow can visualize the phenotype and track clonal evolution in AML

## Graphical abstract

**Figure:**
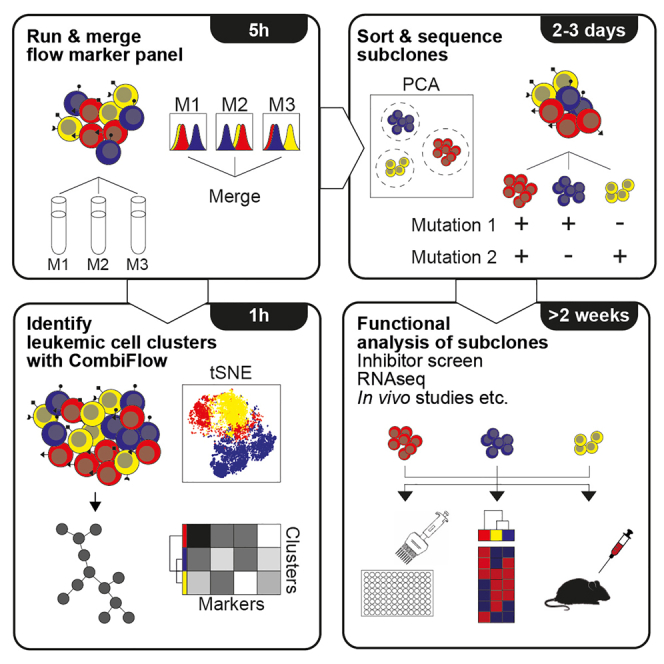

## Teaser

Many cancers, including leukemias, are dynamic oligoclonal diseases. Tools to identify and prospectively isolate genetically distinct clones for functional studies are needed. We describe our CombiFlow protocol, which is a combinatorial flow cytometry-based approach to identify and isolate such distinct clones. CombiFlow enables the visualization of clonal evolution during disease progression and the identification of potential relapse-inducing cells at MRD time points. The protocol can be adapted to various research questions and allows functional studies on live sorted cell populations.

## Before you begin

The protocol below describes the analysis of two diagnosis samples, a paired relapse sample, and a healthy control sample. The protocol may also be applied to the comparison of multiple diagnostic samples within a subgroup of AML or to longitudinally track disease progression by including data from MRD time points. Preparation consists of:

1. Installation of the required R packages (key resources table) for the CombiFlow analysis
2. Primer design for targeted sequencing.

## Key resources table

**Table:** 

REAGENT or RESOURCE	SOURCE		IDENTIFIER
**Antibodies**			

ADAM17-PE (10 μL∗∗)	R&D Systems		Cat#FAB9301P RRID:AB_357217 Clone 111633
ALCAM-PE∗ (1 μL)	BioLegend		Cat#343904 RRID: AB_2289302 Clone 3A6
CCR1-PE (5 μL)	Miltenyi Biotec		Cat#130-100-367 RRID:AB 2655839 Clone REA158
CD117-PE (3 μL)	BD Biosciences		Cat#555714 RRID: AB_396058 Clone YB5.B8
CD123-PE∗ (3 μL)	BioLegend		Cat#306006 RRID: AB_314580 Clone 6H6
CD151-PE∗ (1 μL)	R&D Systems		Cat#FAB1884P RRID: AB_2228965
CD19-PE (5 μL)	BD Biosciences		Cat#345777 RRID: AB_2868805 Clone 4G7
CD25-PE∗ (3 μL)	BioLegend		Cat#302606 RRID: AB_314276 Clone BC96
CD200-PE∗ (5 μL)	R&D Systems		Cat#FAB27241P RRID: AB_1061611 Clone 325516
CD206-PE (5 μL)	R&D Systems		Cat#FAB25342P RRID: AB_10889015
CD274-PE (3 μL)	BioLegend		Cat#329706 RRID: AB_940368 Clone 29E.2A3
CD34-APC (backbone, see 9)	BD Biosciences		Cat#555824 RRID: AB_398614 Clone 581
CD38-FITC (backbone, see 9)	BD Biosciences		Cat#555459 RRID: AB_395852 Clone HIT2
CD45-PE-Cy7 (backbone, see 9)	BD Biosciences		Cat#557748 RRID: AB_396854 Clone HI30
CD45RA-BV421 (backbone, see 9)	BioLegend		Cat#304108 RRID: AB_314412 Clone HI100
CD47-PE∗ (3 μL)	BD Biosciences		Cat#556046 RRID: AB_396317
CD82-PE∗ (1 μL)	BioLegend		Cat#342104 RRID: AB_1595455 Clone ASL-24
CD93-PE (3 μL)	BioLegend		Cat#336108 RRID: AB_2076048 Clone VIMD2
CD97-PE∗ (1 μL)	BioLegend		Cat#336308 RRID: AB_2076182 Clone VIM3b
CD99-PE∗ (1 μL)	BioLegend		Cat#371306 RRID: AB_2616974 Clone 3B2/TA8
CLL1-PE∗ (5 μL)	R&D Systems		Cat#FAB2946P RRID: AB_10892515
CXCR4-PE (3 μL)	BioLegend		Cat#306506 RRID: AB_314612 Clone 12G5
EMR2-PE (1 μL)	Miltenyi		Cat#130-119-810 RRID: AB_2751862 Clone REA302
ESAM-PE (5 μL)	R&D Systems		Cat#FAB4204P RRID: AB_2293790 Clone 408519
ESEL-Fc-PE (1 μL)	GlycoMimetics		Cat#B252284
FLT3-PE∗ (10 μL)	BD Biosciences		Cat#558996 RRID: AB_397175
GPR56-PE∗ (1 μL)	BioLegend		Cat#358204 RRID: AB_2562084 Clone CG4
IFNGRI-PE∗ (1 μL)	R&D Systems		Cat#FAB673P RRID: AB_2264548
IL1RAP-PE∗ (3 μL)	R&D Systems		Cat#FAB676P RRID: AB_10717521
IL6-PE (1 μL)	R&D Systems		Cat#FAB227P RRID: AB_2127909 Clone 17506
ITGA5-PE∗ (3 μL)	BD Biosciences		Cat#555617 RRID: AB_395984
ITGA6-PE (3 μL)	BioLegend		Cat#313612 RRID: AB_893373 Clone GoH3
ITGAE-PE (3 μL)	BioLegend		Cat#350206 RRID: AB_10641843 Clone Ber-ACT8
ITGB7-PE (5 μL)	R&D Systems		Cat#FAB4669P RRID: AB_1964636 Clone 473207
JAMC-PE (5 μL)	R&D Systems		Cat#FAB11891P RRID: AB_2128938 Clone 208212
LILRB2-PE (5 μL)	Miltenyi Biotec		Cat#130-100-564 RRID: AB_2659347 Clone REA184
Propidium iodide	Invitrogen		Cat#P3566
PVR-PE (5 μL)	R&D Systems		Cat#FAB25301P RRID: AB_2269068 Clone 300907
SEMA4D-PE (10 μL)	R&D Systems		Cat#FAB74701P RRID: AB_2732844 Clone 758726
SIRPA-PE (10 μL)	Thermo Scientific		Cat#MA1-74228 RRID: AB_1955117 Clone 15-414
TGFBRII-PE (10 μL)	R&D Systems		Cat#FAB241P RRID: AB_2044728 Clone 25508
TIM3-PE∗ (3 μL)	BioLegend		Cat#345006 RRID: AB_2116576 Clone F38-2E2

**Biological samples**			

AML mononuclear cells	UMCG		n/a

**Chemicals, peptides, and recombinant proteins**			

iQ SYBR Green Supermix	Bio-Rad		Cat#170-8887
FcR blocking reagent	Miltenyi Biotec		Cat#130-059-901
DAPI	Thermo Scientific		Cat#D1306
Bovine Serum Albumin fraction V (BSA)	Roche		Cat#10735094001
Calcium chloride dihydrate	Merck Millipore		Cat#10035-04-8
Heparin	Hospital pharmacy UMCG		Cat#EP00242
MgSO_4_	Sigma-Aldrich		Cat#M2643-500G
DNase I	Sigma-Aldrich/Roche		Cat#112854932001

**Critical commercial assays**			

TruSight™ Myeloid Sequencing Panel	Illumina		Cat#FC-130-1010
NucleoSpin Tissue Kit	MACHEREY-NAGEL		Cat#740952

**Deposited data**			

Marker panel – histograms R script	This paper		n/a
CombiFlow R script	This paper		n/a
Tutorial videos	This paper		n/a

**Oligonucleotides**			

gDNA primers for mutation detection	de Boer et al. (2018)		n/a

**Software and algorithms**			

FlowJo™ Software VX		Becton, Dickinson and Company	https://www.flowjo.com/
R, version 4.0.2		R Foundation for Statistical Computing	https://www.r-project.org/
RStudio®		RStudio	https://www.rstudio.com/
Infinicyt™		Cytognos	https://www.cytognos.com/infinicyt/
Chromas Lite		Technelysium Pty Ltd	https://technelysium.com.au/wp/chromas/
Clone Manager		Sci-Ed Software	https://www.scied.com/dl_cm10.htm
Adobe Illustrator		Adobe	https://www.adobe.com/nl/products/illustrator.html

**Other**			

NCBI gene database		National Center for Biotechnology Information	https://www.ncbi.nlm.nih.gov/gene/
Hanks’ Balanced Salt Solution (HBSS)		Gibco	Cat#14170-088
Trypan blue		Sigma-Aldrich	Cat#T6146
Fetal bovine calf serum		Sigma-Aldrich	Cat#F7524
Newborn calf serum		Gibco	Cat#26010-074
15 mL Centrifugal tubes		Greiner Bio-One	Cat#188271
12 × 75 Polystyrene tube		Greiner Bio-One	Cat#120180
Cryovials		Greiner Bio-One	Cat#1222643

∗ These are the markers we prioritize in case of limited patient material.

∗∗ Volume per tube of 100 μL

## Materials and equipment

**Table:** 

ESEL-Fc buffer	Final concentration	Stock concentration	Amount
HBSS	n/a	n/a	49.5 mL
CaCl_2_	1 mM	100 mM	0.5 mL
BSA	0.5%	n/a	0.25 g
**Total**	**n/a**	**n/a**	**50 mL**

Store at 4°C.

**Table:** 

NCS mix	Final concentration	Stock concentration	Amount
NCS	n/a	n/a	6 mL
Heparine	5 U/mL	1000 IU/mL	500 μL
MgSO_4_	4 μM	200 mM	500 μL
DNase I	20 U/mL	1 mg/mL	500 μL
**Total**	**n/a**	**n/a**	**7,5 mL**

Make fresh each time.

**Table:** 

R Package	Reference
CATALYST	(Crowell, 2021)
ConsensusClusterPlus	(Wilkerson and Hayes, 2010)
cowplot	(Wilke, 2020)
devtools	(Wickham, 2021a)
dplyr	(Wickham, 2021b)
emstreeR	(Quadros, 2020)
factoextra	(Kassambara, 2020b)
FactoMineR	(Le and Husson, 2008)
flowCore	(Ellis, 2021)
FlowSOM	(Van Gassen et al., 2015)
flowWorkspace	(Finak, 2021)
fpc	(Hennig, 2020)
ggcyto	(Van et al., 2018)
ggplot2	(Wickham, 2016)
ggpubr	(Kassambara, 2020a)
ggrepel	(Slowikowski, 2021)
ggsci	(Xiao, 2018)
gridExtra	(Auguie, 2017)
igraph	(Csardi, 2006)
limma	(Ritchie et al., 2015)
lme4	(Bates et al., 2015)
magrittr	(Bache, 2020)
MASS	(Venables and Ripley, 2002)
matrixStats	(Bengtsson, 2021)
mclust	(Scrucca et al., 2016)
multcomp	(Hothorn et al., 2008)
mvtnorm	(Genz, 2020)
pheatmap	(Kolde, 2019)
purrr	(Henry, 2020)
RColorBrewer	(Neuwirth, 2014)
RcppArmadillo	(Eddelbuettel and Sanderson, 2014)
readr	(Wickham, 2020)
readxl	(Wickham, 2019a)
reshape2	(Wickham, 2007)
RJSONIO	(Temple Lang, 2020)
robustbase	(Maechler, 2021)
Rtsne	(Krijthe, 2015)
tidyverse	(Wickham, 2019b)
umap	(Konopka, 2020)
viridis	(Garnier, 2021)
XML	(Temple Lang, 2021)

## Step-by-step method details

### Thawing an AML sample

**Timing: 45 min–1 h**

AML samples (bone marrow or peripheral blood) stored in liquid nitrogen can be thawed and immediately stained with antibodies for the marker panel or cell sorting. The used thawing procedure, as previously described (Schuringa and Schepers, 2009; van Gosliga et al., 2007), consists of the following steps:

1. Thaw two tubes of 6 mL newborn calf serum (NCS)
2. Collect the AML sample from the liquid nitrogen storage and quickly thaw the sample in a water bath kept at 37°C
3. Transfer the AML sample to an NCS tube and centrifuge for 5 min at 450 *g*
4. In the meantime, prepare NCS mix by adding 5 U/mL heparine, 4 μM MgSO_4_ and 20 U/mL DNAse I to the other NCS tube
5. Resuspend the AML cell pellet in the pre-warmed NCS mix
6. Incubate the cells at 37°C for 15 min
7. Spin down cells and resuspend in PBS-mix (6 mL PBS + DNAse (5 U/mL) and MgSO_4_ (4 μM))
8. Count the viable cells using Trypan Blue and continue with:
  a. Run the marker panel or
  b. Sorting of potential subclones

**CRITICAL:** 30–50% of the AML cells will not survive the thawing process. To obtain a higher yield speed is essential in steps 2 and 3. Complete steps 2 and 3 within 15 minutes. If less than 50% viable cells are obtained, it can be considered to perform a Ficoll separation to remove deal cells.

### Run the marker panel

**Timing: 4 h**

Plasma Membrane Protein marker expression data is acquired by flow cytometry that can be used as input for the following analyses: 1) Leukemic cells can be identified based on aberrant expression of one or more markers compared to the healthy control. 2) Aberrantly expressed plasma membrane protein marker expression can be used to identify and sort potential subclones. 3) Disease progression can be tracked by running the panel on longitudinal samples from individual patients.

9. Cells are stained for markers CD34, CD38, CD45 and CD45RA, from now onwards called backbone stain. These markers are used as a basis to merge all measured plasma membrane protein markers together and are therefore present in every tube. More detailed explanation about this principle can be found in **Merge the marker panel flow data.** Addition of antibodies can be performed at 15°C–25°C. All stainings are carried out at 4°C in the dark.
  a. Label unstained and single stained control tubes
  b. Take 15 million viable mononuclear cells (MNCs) of the thawed AML sample and resuspend this in 320 μL PBS
  c. Add 2 μL of cell suspension to the unstained and single stained control tubes containing 98 μL PBS
  d. Add the backbone stain to the remaining cells:
    i. FcR blocking reagent 10 μL
    ii. CD34-APC 9 μL
    iii. CD38-FITC 9 μL
    iv. CD45-PECy7 3 μL
    v. CD45RA-BV421 3 μL
10. Stain cells for 20 min at 4°C in the dark
  a. Label tubes for the marker panel (1-36)
  b. Add PBS to the backbone-stained cells in a total volume of 100 μL ∗ number of markers
  c. Add 100 μL of backbone-stained cells to each of the labeled tubes. Resuspend in between so that all tubes have a similar amount of cells
  d. Add one PE-labeled plasma membrane protein marker antibody per tube. The volume of antibody per tube is indicated in the key resources table.
  e. Stain cells for 30 min at 4°C in the dark
  f. Wash cells with 2 mL of PBS per tube and centrifuge at 450 *g* for 5 min
  g. Remove PBS and resuspend cells in 100 μL fresh PBS
11. Acquire expression data using a flow cytometer

***Note:*** The extra volume of backbone-stained cells will be approximately 300 μl. Keep this at 4°C until all PE-labeled marker panel antibodies have been added. It can serve as back-up if a pipetting mistake is made with the marker panel.

***Note:*** The steps described above have been optimized for staining of cells in FACS tubes. For staining of cells in a 96-well plate some adjustments may be needed in the protocol.

***Note:*** While we have used a backbone panel with antibodies against CD34, CD38, CD45 and CD45RA, other markers, or additional markers such as CD64 or CD117, can be included as backbone markers as well.

***Note:*** The percentage of viable, thawed cells when measuring the marker panel usually lies between 70-90%. The largest cell loss during the thawing procedure is cleaned by the DNase in the NCS mix and does not result in the accidental staining of large number of dead cells. If desired, PI (at 1 μg/mL) can be added to the backbone panel in order to discriminate viable cells. In case of limited patient material, for instance at follow-up time points, a prioritized list consisting of 15 markers can be used, indicated by an asterisk behind the antibody in the key resources table. The ranking of these markers was based on the number of times it was positive in a cohort of 87 AML samples and on literature describing the use of specific markers in AML.

***Note:*** We acquire flow data at the MacsQuant Analyzer 10. Other flow cytometers may also be used if the lasers are sufficient for the used fluorophores.

***Optional:*** Experiment can be paused here. Data analysis and the thawing of a second AML sample for sorting can be done on a different day**.**

### Merge the marker panel flow data

**Timing: 30 min**

A limiting factor in flow cytometry is that the number of markers that can be measured per tube is limited due to availability of fluorochrome-labeled antibodies with distinct excitation and emission spectra and flow cytometers with multiple lasers. To circumvent this problem, we add a backbone of markers in each independent FACS tube. Additional to the backbone in each of the tubes an antibody against a specific plasma membrane marker is added, that can carry the same fluorochrome, in our case PE. Flow data acquired in separate tubes can be combined based on the expression of the backbone markers using Infinicyt™. With the recent development of full spectrum flow cytometers, such as the Cytek Aurora, more fluorochromes may be included to further increase the resolution and reduce analysis time.

12. Gate the FCS files in FlowJo
  a. Export the cells of interest as new FCS files. Here, we make use of CD45^+^ cells of which 10.000 events were exported per FCS file. See Methods video S1 for stepwise instructions
13. Use Infinicyt™ to combine FlowJo™ exported FCS files into a merged file. See Methods video S2 for stepwise instructions
  a. Within the Infinicyt™ environment, select ‘Calculate data’ in the ‘Guided modules’ window
  b. Load all FCS files necessary for your analysis. For instance, when disease progression is monitored load all FCS files per patient for every time point
  c. Deselect parameters that can be excluded, such as the time parameter HDR-T
  d. Separate the PE parameter so that one row is created for every included marker
  e. Check for a potential merge warning. Check out troubleshooting – problem 1 if this is the case
  f. Rename the rows that were created in step 13d by the corresponding plasma membrane protein marker name
  g. Check the “The selected populations were unequivocally identified” box and select calculate data
  h. Save the calculated data file before continuing to the analysis window
  i. Continue to the analysis window and save
14. Export the merged FCS file
  a. For continuation of CombiFlow, see **CombiFlow**
  b. For identification of potential subclones, see **Identification of potential subclones.** Troubleshooting 1

***Note:*** By making use of Infinicyt to separate the PE-labeled markers, the panel can be easily adapted. The number of markers can be increased, provided that a PE-labeled antibody is available.

***Note:*** Pay attention to the names of the markers and make sure these are consistent throughout analyses. This will avoid errors in the CombiFlow pipeline. Spaces are allowed but will be replaced by an underscore in the Combiflow analysis script.

***Note:*** Additional information on exporting FCS files can be found here: *FCS file export from FlowJo*. Additional explanations on the merging of FCS files and the APS plotting can be found in the Infinicyt documentation available on the Cytognos website.

***Note:*** Select which markers to include in the export if the merged file is used as input for CombiFlow, since here markers should match between included files.

Methods video S1. Export CD45+ cells as new FCS files, related to step 12

Methods video S2. Creating a merged FCS file with Infinicyt, related to step 13

### Identification of potential subclones

**Timing: 30 min–1 h**

**Figure 1:**
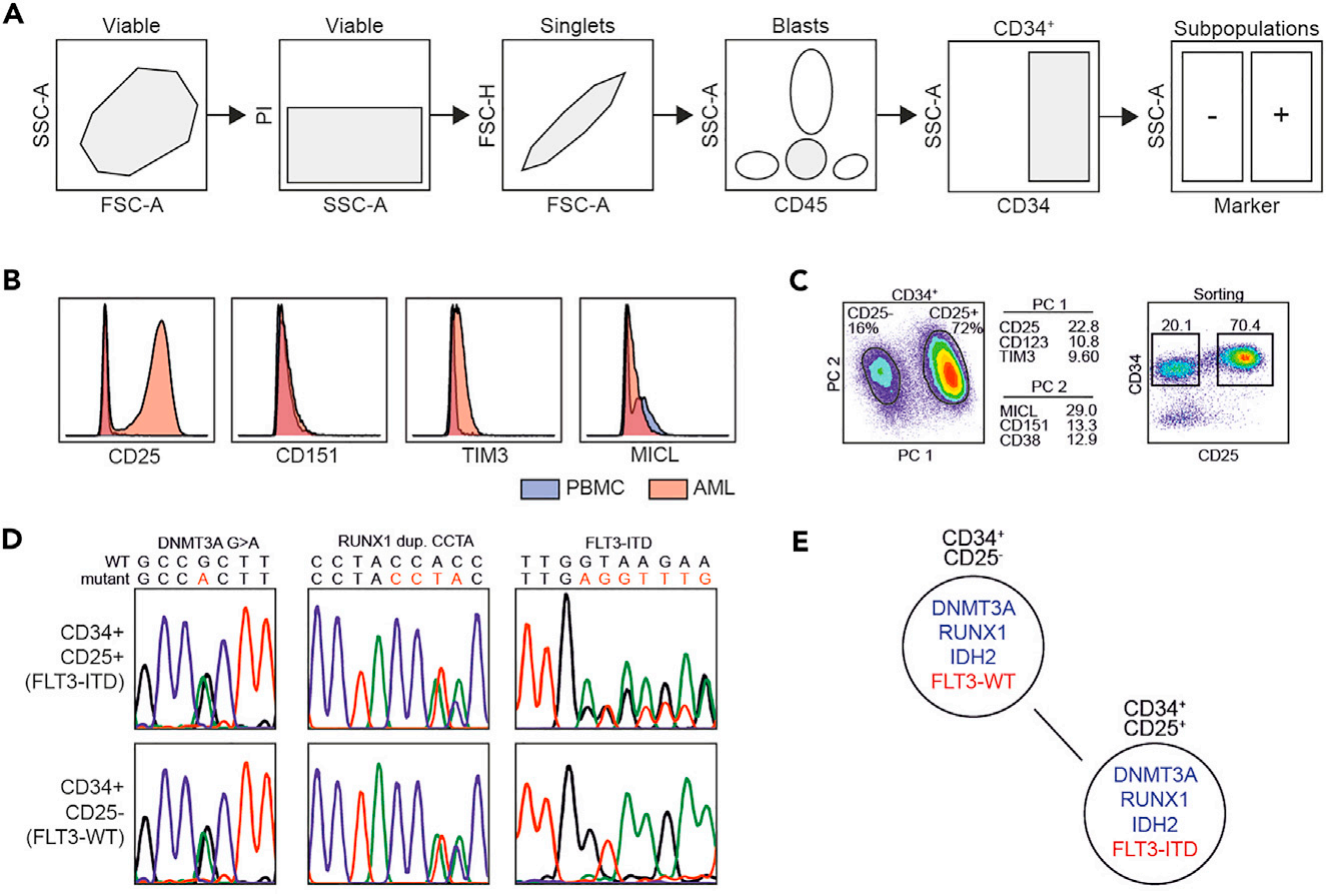
Outcome of subclone identification by Principal Component analysis (A and B) (A) Gating strategy for the sorting of subpopulations (B) Expression of plasma membrane protein markers of an AML sample plotted together with the expression of these markers in a healthy control in order to identify which plasma membrane protein markers are aberrantly expressed. (C) A Principal Component analysis was performed using aberrantly expressed plasma membrane protein markers, showing the most distinguishing markers for Principal Component 1 (PC1) and Principal Component 2 (PC2). Plasma membrane protein marker CD25 was identified as most distinguishing marker. A FACS sort was performed based on CD34 and CD25 expression resulting in two subclones: subclone 1 CD34^+^CD25^+^ and subclone 2 CD34^+^CD25^-^. (D) To identify whether the sorted subclones were genetically distinct targeted sequencing was performed for the mutations DNMT3A G>A, RUNX1 duplication CCTA and FLT3-ITD. It was found that both subclones have a DNMT3A and RUNX1 mutation, but only subclone 1 CD34^+^CD25^+^ also has a FLT3-ITD. (E) Pedigree of the subclones depicting the possible evolution pattern. This figure was adjusted from de Boer et al., 2018; figure 3.

With the merged flow data from **Merge the marker panel flow data** marker expressions can be compared with marker expressions of a healthy donor. Aberrantly expressed markers can be selected and used for further analysis in the Infinicyt™ environment to identify potential subclones as depicted in Figure 1. First, markers with aberrant expression are selected and used as input for the Principal Component analysis, which is used to identify the most discriminating marker (Figures 1A and B). Subpopulations can be sorted based on this marker and sequenced (Figure 1C). Based on the sequencing outcomes, a pedigree can be created that depicts the expected evolutionary track of the leukemic cells (Figure 1D).

15. Prepare the data from the merged file for further analysis
  a. Export marker expression data per cell from the population of interest in the merged FCS file using FlowJo™
  b. Prepare a data file for the healthy control
16. Run R script: “Marker panel – histograms” to plot plasma membrane protein marker expression data of a healthy control and the data of interest to find aberrantly expressed proteins
  a. Save generated histogram plot as pdf
17. Select plasma membrane protein markers that are aberrantly expressed in comparison to the healthy control using the previously generated histogram plots
18. Open the analysis file, saved in step 13i, in the Infinicyt™ environment. See Methods video S3 for stepwise instructions
  a. If necessary: gate population of interest
  b. Check if Negative Visibility Configuration is selected for Automatic Negativity
19. Perform a Principal Component analysis
  a. Plot an APS (Automated Population Separator) diagram (Ctrl+D)
  b. Deselect backbone markers and select aberrantly expressed markers (Right click: Diagram Configuration → Parameters Configuration)
  c. Obtain Principal Component values (Ctrl+I) and select most distinguishing markers
  d. Gate subpopulations and visualize in dot plots
20. Export all graphs and individual subpopulations for use in Manuscript Figures or downstream analysis
21. Determine a sorting strategy using the most distinguishing marker(s) in the merged FCS file using Flow Jo™
  a. Continue with Sorting of potential subclones

***Note:*** If more than one plasma membrane protein marker is needed to distinguish between potential subclones, it is necessary to check whether this marker is available with a different fluorophore than PE before continuing with **Sorting of potential subclones**. Troubleshooting 2

Methods video S3. Identification of potential subclones, related to steps 18 and 19

### Sorting of potential subclones

**Timing: 4 h**

After determining the most distinguishing plasma membrane protein marker(s) to identify potential subclones using the merged FCS file and Infinicyt™. The potential clones can be sorted using the protocol below. This part is a continuation of **Thawing an AML**.

22. To sort potential subclones AML cells need to be stained with part of the backbone markers and the most distinguishing plasma membrane protein markers (PMPs) that were determined by a Principal Component analysis
  a. Label tubes for unstained and single stained controls
  b. Resuspend all thawed AMLs cells in PBS-mix (10 × 10^6^ cells/100 μL)
  c. Add 1 ul of cells to unstained and single stained controls containing 99 μL of PBS-mix
  d. Add staining to the remaining cells. Volume/100 μL.
    i. Fc block 3 μL
    ii. CD34-APC 3 μL
    iii. CD45-PECy7 1 μL
    iv. PMP marker 1-PE
    v. PMP marker 2 (optional)
  e. Stain cells for 20 min at 4°C
23. In the meantime, prepare collection tubes containing medium with FCS
24. Wash cells with PBS and resuspend them:
  a. For unstained/single cell tubes in ±300 μL PBS-mix
  b. For main tube in 1000 μL PBS-mix per 50 × 10^6^ cells
  c. Add PI stain to the main tube at 1 μg/mL
25. Sort subclones of interest. If the FACS sorter is clogged, check troubleshooting – problem 2 for extra instructions
26. Process sorted cells for downstream analysis e.g., targeted sequencing, RNA sequencing or culturing

***Note:*** This sorting protocol is optimized for the Beckman Coulter MoFlo Astrios and XDP at our FACS facility. Before starting sorting subclones it is advised to optimize the protocol for your FACS equipment. Troubleshooting 3 and 4

### CombiFlow

**Timing: 1 h**

**Figure 2:**
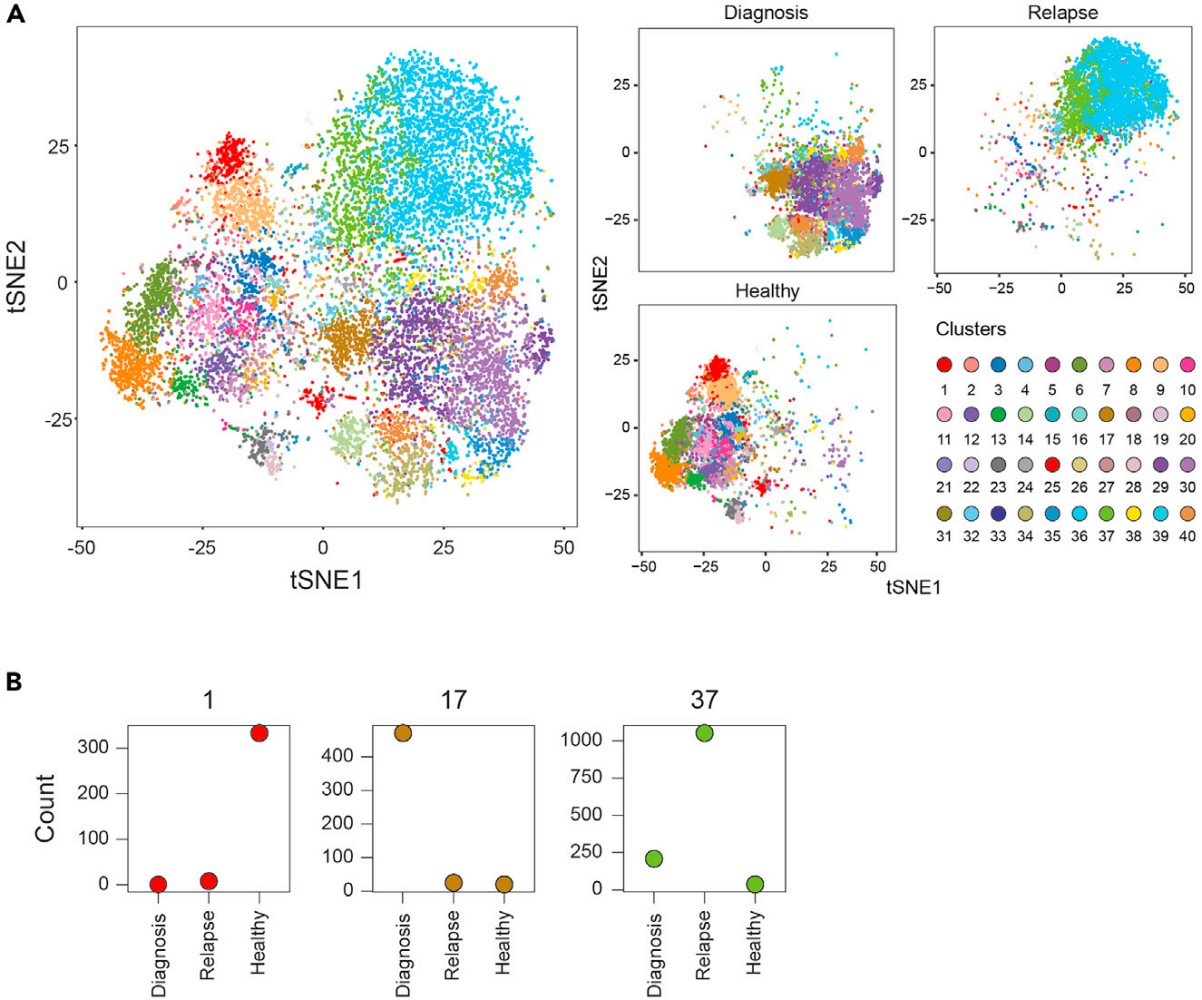
Example of assigning clusters to a sample (A) Total tSNE landscape of combined samples (left) and tSNE landscape per sample (right) colored by forty clusters. (B) Cell count per cluster per sample. Clusters were assigned to diagnosis, MRD, relapse, or healthy indicated in bold.

**Figure 3:**
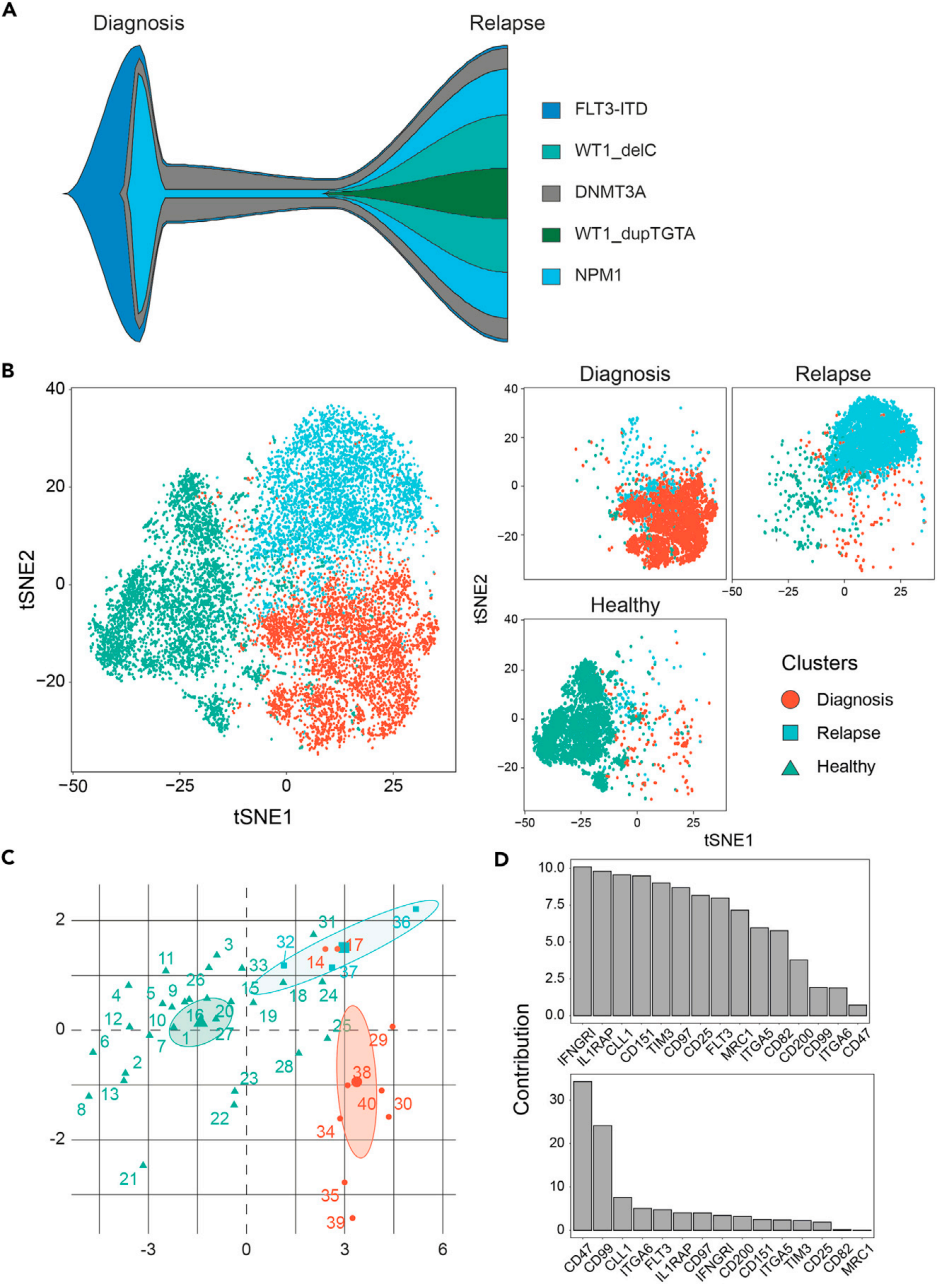
Outcome of CombiFlow pipeline for a patient with paired diagnosis and relapse samples and a healthy control (A) TruSight sequencing Variant Allele frequencies at diagnosis and relapse depicted in a fish plot. (B) tSNE landscape per sample. Distinction between diagnosis (red), relapse (blue) and healthy (green). (C) Principal Component analysis of the forty clusters, colored by sample. (D) Ranking of markers contributing to Principal Component 1 (PC1, top) or Principal Component 2 (PC2, bottom).

The CombiFlow pipeline can be used to study the hematopoietic landscape of healthy and leukemic samples at diagnosis and longitudinally. Potential relapse-inducing populations can be identified at MRD time points and changes in clonal composition can be monitored based on marker expression data as depicted in Figures 2 and 3. The CombiFlow pipeline is based on an analysis workflow for CyTOF data (Nowicka et al., 2017). Whereas in the described examples clusters are assigned to visualize differences between diagnosis, relapse and healthy (Figure 2), it may also be used to visualize different cell types such as blasts, lymphocytes and mature myeloid cells.

27. Data preparation
  a. Export population of interest from the merged FCS files using FlowJo™ and zip the files
  b. Create a metadata file
  c. Create a panel file
28. Create a cluster file. The number of clusters can be adjusted based on the expected number of populations. If this is not known before running the analysis, a fixed number of clusters can be used. Here, 40 clusters were created per analysis to avoid missing out on smaller, more rare cell populations
29. Clusters are formed using the FlowSOM algorithm (Van Gassen et al., 2015) and can be visualized for all samples or per sample using tSNE landscapes. These can be colored by marker expression to identify and visualize specific cell populations such as CD34^+^ cells or CD4^+^ lymphocytes
30. Create a heatmap that depicts the transformed marker expression per cluster. This can be used to assign clusters to specific cell types
31. Run the script until the step ‘Manual clustering per sample’
  a. Clusters can be assigned to a sample based on the cluster cell count per sample
  b. Create a sample file
32. Visualize the newly assigned clusters for all samples combined or per sample
33. A new heatmap can be created, which depicts the combined expression of all clusters assigned to one specific sample
34. Continue running the script until the Principal Component analysis step
  a. Select which markers and clusters to include in the Principal Component analysis. A Principal Component analysis can be performed for all included samples, but a comparison of diagnosis and relapse, without the healthy clusters, is also a possibility
  b. Run the Principal Component analysis to identify the most discriminating marker(s). Troubleshooting 5

***Optional:*** Clusters can be assigned to a sample but also cell type. In order to do so, create a file similar to the cluster and sample file but now assign each cluster to a cell type based on the expression data in the heatmap. For example, a cluster with small size, high CD45 and CD19 expression may be assigned to either lymphocytes or, more specifically, B cells.

***Note:*** Some clusters may have cells originating from both the leukemic and healthy sample. We opted to assign these cells to the healthy sample until the ratio Healthy/Leukemic cells fell below 1:8.

***Note:*** Uniform manifold approximation and projection (UMAP) visualization may be an alternative for the currently used tSNE plots. Implementation of UMAP into the CombiFlow pipeline is currently ongoing, as well as a more automated, machine-learning approach for the cluster assignments to reduce analysis time.

## Expected outcomes

With this protocol we generated marker expression profiles of 2 diagnosis samples, a paired relapse sample and a healthy control. Of the unpaired diagnosis sample we selected the plasma membrane protein markers that were aberrantly expressed in comparison to the expression levels in the healthy control (Figure 1A). Using the aberrantly expressed markers a Principal Component analysis was performed in the Infinicyt™ environment and the marker CD25 was identified as most distinguishing maker (Figure 1B). The diagnosis sample was sorted in two populations: CD34^+^CD25^+^ and CD34^+^CD25^-^, and cells were processed for further analysis. We isolated gDNA from both clones and performed targeted sequencing for the mutations DNMT3A G>A, RUNX1 dup. CCTA and FLT3-ITD. We found that DNTM3A and RUNX1 were present in both clones, but FLT3-ITD was only found in the CD25^+^ clone (Figure 1C). Based on these findings we can predict a possible evolutionary pattern of the clones found in this particular individual. Various downstream analyses were performed on the sorted subclones. RNA sequencing and GO analysis revealed enrichment of processes related to cell proliferation, mitochondrial activity and cytokine signaling in the FLT3-ITD clone, whereas the FLT-WT clone was enriched for processes related to chromatin organization and histone modification. By *in vitro* culture of the subclones followed by treatment with the FLT3 tyrosine kinase inhibitor AC-220, differences in sensitivity to AC-220 were found as only the FLT3-ITD subclone showed reduced cell counts and increased levels of apoptosis.

The CombiFlow examples show one AML patient with diagnosis and relapse samples and a healthy control. In Figure 2 the forty clusters are visualized in total and per sample. Here it can be observed that the diagnosis and relapse differ based on their marker expression profiles. TruSight sequencing data was also available at diagnosis and relapse, variant allele frequencies at both time points are depicted in a fish plot (Figure 3A). Clonal evolution between diagnosis and relapse was observed as the BCOR mutation was no longer detected at relapse, whereas the mutations in KMT2A and CEBPA newly appeared. The size of the NPM1 mutated clone increased. These genetic differences between diagnosis and relapse were visualized in the tSNE landscape (Figure 3B), indicating that the markers used to create the landscape could separate cell clusters derived from the diagnosis and relapse sample. The healthy control clustered away from the AML samples. The Principal Component analysis reflected this separation and identified ITGA5, CD97 and IL1RAP as the top markers to distinguish between the AML and healthy cells (Figures 3C and 3D). CD47, CD99 and CLL1 differ between diagnosis and relapse and possibly within the healthy cells as well (Figure 3D).

## Limitations

A possible bottleneck is the merging of the flow data in Infinicyt™. This is sensitive to aberrations in expression of the backbone markers. It may lead to having to exclude one of the markers. Furthermore, it is possible that the subclone that was found is very small, so there are not enough cells left after sorting for further analysis. In addition, differences in mutational status are not always found in the sorted subclones, resulting in having to go back to the drawing board. Another limitation is that the number of events that can be included in the CombiFlow analysis is currently relatively low. This is due to a lack of computational power. By running the analysis on a cluster this may be circumvented and the number of events can be increased. Another issue that is commonly present when working with patient material is a low quantity of cells. For longitudinal studies this is mainly at MRD time points, when most of the cells are expected to be healthy but a small aberrant population may remain. A smaller marker panel may be used in these instances to reduce the amount of needed material. Markers may be selected based on their aberrant expression at diagnosis, yet this may exclude markers with a different-from-normal expression that arise following treatment. Lastly, we are currently working on automating the process by a machine-learning approach of creating and assigning clusters to a sample or cell type to reduce analysis time and remove potential user bias.

## Troubleshooting

### Problem 1

A merge warning occurs (step 13).

### Potential solution

Exclude the FCS file that is listed first in the merge warning window or separate the backbone marker from the aberrant file from the rest similar to what is needed for the PE-labeled markers. A disadvantage of this is that the marker can no longer serve as a backbone marker and the calculate data step will take longer. Another possibility is to include more events in the ‘calculate data’ step. For example, instead of including only the blasts or CD45^+^ cells, include all viable cells. This will mainly help if cell numbers per file are relatively low.

### Problem 2

No subpopulations can be identified in the APS plot (step 19).

### Potential solution:

This is a possibility as some AMLs do not have clear subpopulations based on their marker expression profile. It may be that markers currently not included in the panel are capable of distinguishing subpopulations in the AML in question. Another option is that the highest ranking marker does have a larger range in expression than other markers but that lower ranked markers have a more bimodal expression pattern indicative of subpopulations. It is therefore, in these instances, recommended to deselect the one or two highest ranking parameters to see if the APS plot changes.

### Problem 3

Stickiness of AML cells result in clogging of FACS sorter (step 25).

### Potential solution

AML cells can be diluted further in PBS mix; the DNaseI in the mix breaks down free DNA. If this is not sufficient cells can be put through a 35 μm cell strainer.

### Problem 4

The sorted subpopulations do not differ in mutation status (step 26).

### Potential solution

It is possible that sorting an AML into subpopulations based on the highest ranking marker(s) in the PCA does not result in genetically distinct subclones. A potential solution may be to sort the AML again, except this time not based on the highest ranking but on the second or third ranking marker. Another explanation for the lack of difference in mutation status may be that the differences in marker expression are not due to genetic, but due to epigenetic or transcriptomic variation between the subpopulations. This will require more extensive analysis of the sorted subpopulations than NGS sequencing.

### Problem 5

Running the CombiFlow script results in an error (step 29)

### Potential solution

Check if the script refers to the correct files. The metadata file should contain the exact file names that are included in the zipped folder containing the FCS files. The markers included in the analysis should match between each FCS file and between the FCS files and the panel file. Lastly, the CombiFlow script can be used for different comparisons, e.g., AML versus healthy or tracking an AML patient longitudinally. This may affect the description of the condition, which should match between the metadata file and the script.

## Resource availability

### Lead contact

Further information and requests for resources and reagents should be directed to and will be fulfilled by the lead contact, Prof Dr. J.J. Schuringa, j.j.schuringa@umcg.nl

### Materials availability

This study did not generate new unique reagents.

## Data Availability

The videos and code generated and used in this study are available at https://github.com/rhoutsma/CombiFlow.
